# Plastomes of Ten *Blumea* Species (Asteraceae): Comparative Analysis and Phylogenetic Relationships

**DOI:** 10.1002/ece3.74361

**Published:** 2026-10-12

**Authors:** Yuemei Zhao, Weixiang Yang, Changjiang Qian, Tao Zhou, Xiao Zhang, Xiaodan Chen, Rongxiang Zhang, Xun Liu

**Affiliations:** ^1^ School of Biological Sciences Guizhou Education University Guiyang Guizhou China; ^2^ College of Coastal Agricultural Sciences Guangdong Ocean University Zhanjiang Guangdong China; ^3^ School of Pharmacy Xi'an Jiaotong University Xi'an Shaanxi China; ^4^ Xi'an Botanical Garden of Shaanxi Province Institute of Botany of Shaanxi Province Xi'an Shaanxi China; ^5^ College of Life Science Shanxi Normal University Taiyuan Shanxi China

**Keywords:** *Blumea*, chloroplast genome, comparative analysis, phylogenetic relationship, positive selection, repeat

## Abstract

*Blumea* (Asteraceae) comprises about 100 species distributed across tropical Asia, Africa, and Australia, many of which have medicinal value. However, morphological variation caused by hybridization has left the taxonomy of this genus unresolved. In this study, we assembled the chloroplast genomes of six *Blumea* species ( *B. repanda* , *B. axillaris* , *B. sinuata* , *B. martiniana*, *B. megacephala* , and *B. formosana* ) and compared them with those of four published species (*B. oxyodonta*, *B. tenella* , *B. aromatica*, and *B. balsamifera* ). The six new plastomes are similar in size (150,783–151,281 bp), with a typical quadripartite structure, each containing 131 genes (86 protein‐coding genes, 37 tRNAs, and 8 rRNAs). Comparative analyses revealed conserved genome architecture, stable IR/SC boundaries, and no rearrangements. Six non‐coding and four coding regions were identified as candidate DNA barcodes. A total of 395 dispersed repeats, 79 tandem repeats, and 547 SSRs were identified. Among the 59 preferred codons, 29 were high‐frequency (RSCU > 1), and 28 of these ended in A/U. Additionally, positive selection was detected in 21 genes, for example, *ycf1*, *ycf2*, *rpoB*, and *ndhA*. Phylogenetic analyses (ML and BI) based on 23 taxa (21 Inuleae species plus two outgroups) showed that *Blumea* is a highly supported monophyletic group (BS = 100%, PP = 1.00), internally divided into three subclades: subclade I ( *B. axillaris* , *B. oxyodonta*, *B. megacephala* , *B. repanda* , *B. sinuata* , and *B. tenella* ), subclade II ( *B. balsamifera* ), and subclade III (*B. aromatica*, *B. formosana* , and *B. martiniana*), with the latter three species all belonging to Section Macrophylla. These genomic resources lay a foundation for future phylogenetic, population‐genetic, and biotechnological studies of *Blumea*.

## Graphical abstract

Comparative analysis of ten *Blumea* plastomes revealed conserved genome architecture and identified mutation hotspots and positively selected genes. Phylogenetic reconstruction clarified the genus's position within Inuleae and supported its monophyly. This study provides valuable genomic resources for *Blumea*.

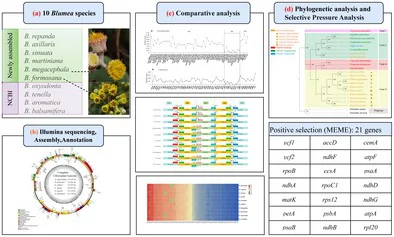

## 1 Introduction

*Blumea* is considered the largest genus in Inuleae (Asteraceae) (Anderberg 1991; Anderberg and Eldenäs 2007), with around 100 species spread across tropical Asia, Africa, and Australia, ranging from herbs, shrubs, and small trees (Anderberg and Eldenäs 2007; Randeria 1960; Bremer 1994; Pornpongrungrueng et al. 2016). This genus is characterized by disciform capitula, a single large oxalate crystal within the cells of the cypsela epidermis, radial thickening of the anther endothecium, and the presence of tailed anthers (Pornpongrungrueng et al. 2016). However, the classification of *Blumea* is complex because of its heterogeneous traits and distinctive characteristics (Randeria 1960; Pornpongrungrueng et al. 2007, 2016; de Candolle 1836; Dakshini and Singh 1978). Over the years, researchers have turned to plastid markers (*trnL‐F, psbA‐trnH*) and nuclear loci (*ITS*, *5S‐NTS*) (Zhang, Yuan, et al. 2019; Pornpongrungrueng et al. 2007, 2009) to sort out its evolutionary relationships; however, its affinities with allied genera remain unresolved due to low substitution rates and complex interspecific relationships. In China, *Blumea* is classified into six sections (Sect. Dissitiflorae; Sect. Macrophillae; Sect. Oxyodontae; Sect. Paniculatae; Sect. Sagittatae; Sect. Semivestitae), based on morphological characteristics (Editorial Committee of Flora of China of Chinese Academy of Sciences 1979).

In China, more than half of the *Blumea* species have documented medicinal and ethnobotanical uses (Jia and Li 2005). Among them, *B. balsamifera* (known locally as Dafeng'ai) has been used for thousands of years across China and other Southeast Asian countries to treat conditions including eczema, dermatitis, beriberi, menorrhagia, and skin injuries (Jia and Li 2005). Modern pharmacological studies have identified L‐borneol as its primary bioactive constituent. This species also holds a special place in traditional Chinese medicine as the only botanical source of Aipian, a substance used to “clear orifices and invigorate the spirit” (Chinese Pharmacopoeia Commission 2020). Other species—such as *B. megacephala* , *B. riparia* , *B. aromatica*, *B. formosana* , and *B. densiflora* —have likewise demonstrated considerable pharmacological potential (Chinese Pharmacopoeia Commission 2020).

Chloroplasts are essential photosynthetic organelles that typically possess a circular genome of 115–165 kb, which is a conserved quadripartite structure comprising a large single‐copy (LSC) region, a small single‐copy (SSC) region, and two inverted repeats (IRs) in most plants (Sugiura 1992, 1994). Chloroplast (cp) DNA sequences are widely used to investigate evolutionary relationships in plants, owing to their stable genome architecture, predominantly maternal inheritance, lack of recombination, and reduced effective population size (Birky et al. 1983). Advances in next‐generation sequencing have made plastid genome assembly both cheaper and faster, which accelerates the proliferation of phylogenomic studies across diverse taxonomic scales (Iram et al. 2019; Jung et al. 2021; Karimov et al. 2025).

Here, we employed Illumina sequencing to assemble complete plastid genomes for six *Blumea* species— *B. repanda* , *B. axillaris* , *B. sinuata* , *B. martiniana*, *B. megacephala* , and *B. formosana* —and compared them with four previously reported cp genomes (*B. oxyodonta*, *B. tenella* , *B. aromatica*, and *B. balsamifera* ). Our objectives were to (1) present these six newly assembled plastomes, (2) perform a comprehensive comparative analysis of genome structure across all 10 *Blumea* species, and (3) clarify the phylogenetic position of *Blumea* within Inuleae based on plastome‐scale data. This study expands the genomic resources available for *Blumea* and provides new insights into the evolutionary dynamics of the genus.

## 2 Materials and Methods

### 2.1 Plant Materials and DNA Extraction

Healthy, newly harvested leaves were sampled from six species ( *B. megacephala* , *B. formosana* , *B. axillaris, B. sinuata*, *B. repanda* , *B. martiniana*) from Yunnan, China (Table 1), plant material collection was permitted by the local government. Plant species identification was carried out by Yuemei Zhao and Changjiang Qian (the first and third authors of this study), voucher specimens have been placed in the Guizhou Education University Herbarium (GEUH), Guiyang, China (Figure 1). Total genomic DNA was isolated from the silica‐gel‐dried leaf tissue using a modified CTAB protocol (Doyle 1987), DNA purity was evaluated on a 1.0% agarose gel; samples that passed quality control were then used for library construction and 150‐bp paired‐end sequencing on the Illumina HiSeq 2500 platform. Sequencing was conducted by Biomarker Technologies Inc. in Beijing, China.

**TABLE 1:** Genome features of the chloroplast genomes of 10 *Blumea* species.

Species	*B. megacephala* ^a^	*B. formosana* ^a^	*B. axillaris* ^a^	*B. sinuata* ^a^	*B. repanda* ^a^
Locations	23.09° N, 103.63° E	23.18° N, 103.77° E	22.88° N, 103.79° E	22.09° N, 103.63° E	22.57° N, 103.83° E
Filtered reads	28,312,092	27,782,666	29,210,094	24,669,508	34,320,980
Q20/Q30 (%)	98.43/95.53	98.77/96.47	98.71/96.23	98.44/95.61	96.81/91.83
Mean coverage	107.48×	314.22×	310.17×	564.91×	144.71×
Size (bp)/GC content (%)	151,042/37.6	151,281/37.5	151,063/37.5	150,783/37.6	151,118/37.5
LSC (bp)/GC content (%)	82,736/35.7	82,894/35.7	82,777/35.6	82,524/35.7	82,788/35.7
SSC (bp)/GC content (%)	18,418/31.0	18,509/30.9	18,438/30.9	18,355/31.0	18,416/31.0
IRs (bp)/GC content (%)	24,944/43.0	24,939/43.0	24,924/43.0	24,952/43.0	24,957/43.0
Number of total genes	131	131	131	131	131
Number of protein‐coding genes	86	86	86	86	86
Number of tRNA genes	37	37	37	37	37
Number of rRNA genes	8	8	8	8	8
Pseudogene	/	/	/	/	/
GenBank number	PX404820	PX404818	PX404823	PX404822	PX404821

Species	*B. martiniana* ^a^	*B. oxyodonta*	*B. tenella*	*B. aromatica*	*B. balsamifera*
Locations	23.05° N, 103.61° E	/	/	/	/
Filtered reads	37,262,874	/	/	/	/
Q20/Q30	97.13/92.25	/	/	/	/
Mean coverage	122.27 ×	/	/	/	/
Size (bp)/GC content (%)	151,168/37.5	150,997/37.5	150,829/37.6	151,275/37.5	151,170/37.6
LSC (bp)/GC content (%)	82,794/35.7	82,745/35.7	82,478/35.7	82,891/35.7	82,740/35.8
SSC (bp)/GC content (%)	18,476/30.8	18,438/30.9	18,439/30.9	18,510/30.9	18,466/31.1
IRs (bp)/GC content (%)	24,949/43.0	24,907/43.0	24,956/43.0	24,937/43.0	24,982/43.0
Number of total genes	131	130	130	131	131
Number of protein‐coding genes	86	86	86	86	85
Number of tRNA genes	37	36	36	37	37
Number of rRNA genes	8	8	8	8	8
Pseudogene	/	/	/	/	1
GenBank number	PX404819	BK013128	BK013129	NC_069835	NC_077558

^a^ Newly obtained chloroplast genomes in this study.

**FIGURE 1:**
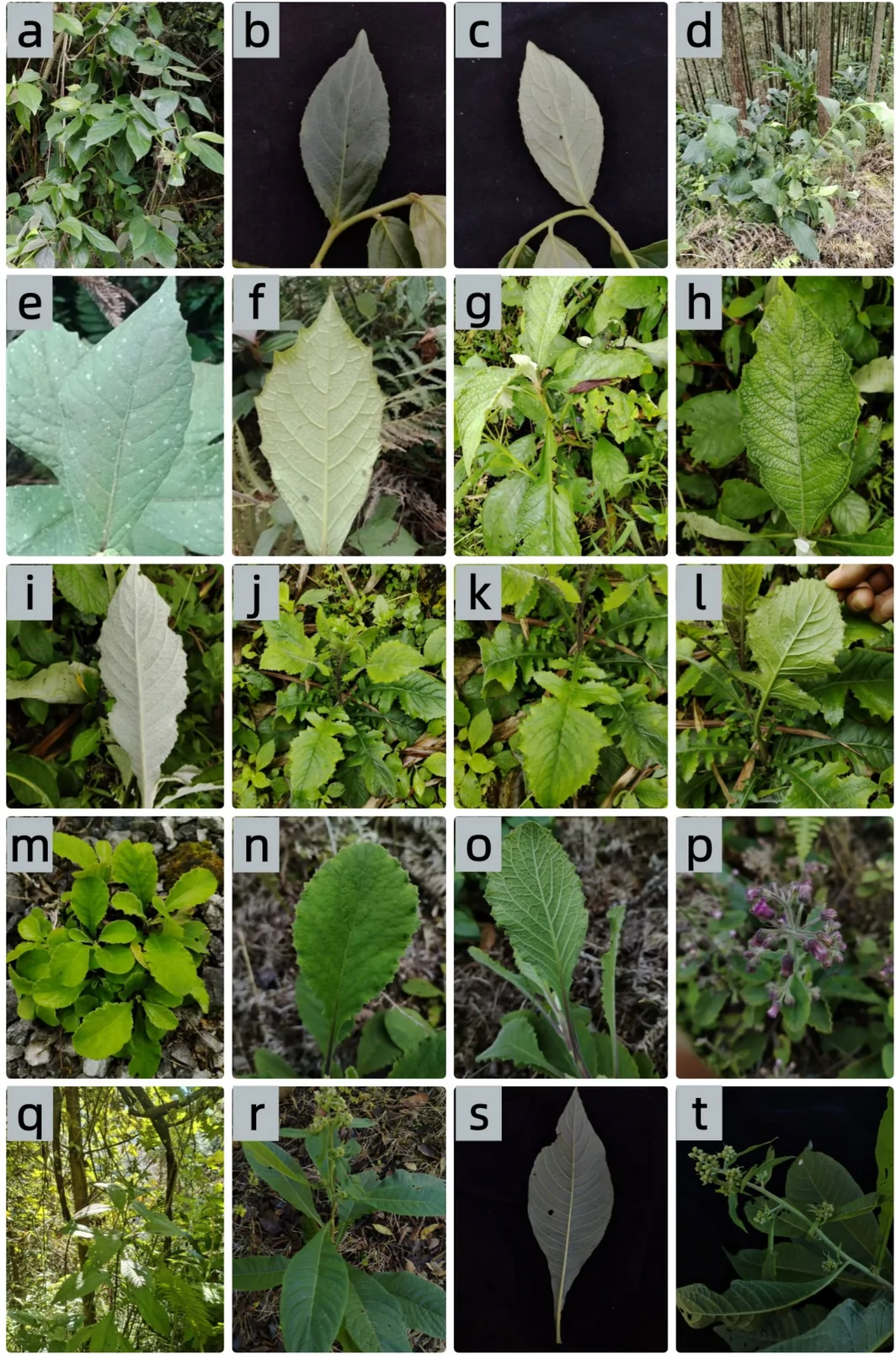
Morphological characteristics of six *Blumea* species in natural habitats (a–c, *B. megacephala* ; d–f, *B. repanda* ; g–i, *B. martiniana*; j–l, *B. sinuata* ; m–p, *B. axillaris* ; q–t: *B. formosana* ).

### 2.2 Illumina Sequencing, Assembly, and Annotation

Low‐quality reads were removed using Fastp v0.23.2 (Chen et al. 2018), and complete chloroplast genomes were assembled with GetOrganelle v1.7.7 (Jin et al. 2020) using the following command: get_organelle_from_reads.py −1 A1.clean.fq −2 A2.clean.fq ‐o A1_output ‐R 10 ‐k 21,45,65,85 ‐F embplant_pt, where A1 and A2 represent the two paired‐end reads of each *Blumea* species. The assembled sequences were then verified by alignment against the reference genome of *Blumea balsamifera* (NC_077558). Initial annotations were performed using CPGAVAS2 (Shi et al. 2019), followed by manual correction in Geneious Prime v2023.2.1 (Kearse et al. 2012). Circular genome maps for the six newly assembled *Blumea* plastomes were generated with OGDRAW (Greiner et al. 2019). These six complete cp genome sequences have been deposited in GenBank under accession numbers PX404818–PX404823 (Table 1). For comparative analysis, four previously published *Blumea* plastomes—*B. oxyodonta* (BK013128), *B. tenella* (BK013129), *B. aromatica* (NC_069835), and *B. balsamifera* (NC_077558)—were retrieved from GenBank and re‐annotated to ensure consistency with the newly generated data (Table 1).

### 2.3 Comparative Plastid Genomic Analysis

To investigate structural variation, we compared IR–SC boundary positions across the 10 *Blumea* plastomes to detect patterns of IR expansion and contraction. Sequence identity among all 10 *Blumea* plastomes was evaluated using mVISTA under the shuffle–LAGAN model (Frazer et al. 2004). Whole‐genome alignments were performed with Mauve v2.4.0 (Darling et al. 2004) to assess genome rearrangement events. Aligned plastomic fragments exceeding 300 bp were extracted, and nucleotide diversity (Pi) was calculated using DnaSP v5.1 (Librado and Rozas 2009).

### 2.4 Microsatellites and Repeated Sequences

Tandem repeats longer than 10 bp were identified using Tandem Repeats Finder (Benson 1999) with parameters set to match = 2, mismatch = 7, indel = 7, a minimum alignment score of 80, and a maximum period size of 500. Dispersed repeats—including forward, reverse, palindromic, and complementary types—were detected using REPuter (Kurtz et al. 2001), requiring a minimum length of 30 bp, sequence identity ≥ 90%, and a Hamming distance ≤ 3. Simple sequence repeats (SSRs) were characterized using MISA (Thiel et al. 2003) with minimum repeat unit thresholds of 10 for mononucleotides, five for di‐ and trinucleotides, and three for tetra‐, penta‐, and hexanucleotides.

### 2.5 Analysis of Codon Usage Bias

Codons serve as the basic units that translate messenger RNA into proteins (Qiu et al. 2011). With 64 possible codons encoding 20 amino acids, those specifying the same amino acid are referred to as synonymous codons. Their usage across genes or genomes is typically non‐random, giving rise to codon bias—a lineage‐specific characteristic shaped by mutation and selection (Grantham et al. 1980). Relative synonymous codon usage (RSCU) quantifies this bias by comparing the observed frequency of a codon to its expected frequency under equal usage among synonymous codons (Sharp and Li 1987). An RSCU value of 1 indicates no bias, while values > 1 or < 1 reflect higher‐ or lower‐than‐expected usage, respectively. In this study, coding sequences (CDS) were extracted and filtered using Perl scripts based on three criteria: (1) presence of a canonical start codon (ATG) and a stop codon (TAA, TAG, or TGA) with no internal termination signals; (2) sequence length ≥ 300 bp; and (3) exclusion of repetitive sequences. For RSCU calculation, stop codons (TAA, TAG, TGA), as well as the codons for methionine (ATG) and tryptophan (TGG)—both of which are encoded by single codons—were excluded, leaving 59 sense codons for downstream analysis. RSCU values for the filtered CDSs were calculated using MEGA 12 (Kumar et al. 2024). Based on the RSCU values of these 59 codons, a heatmap was constructed using TBtools (Chen et al. 2023) to visualize the codon usage variation among the 10 *Blumea* species.

### 2.6 Phylogenetic Analyses and Adaptive Evolution

To reconstruct the phylogeny of *Blumea* and its allies, we assembled a dataset of 10 *Blumea* species and 11 related Inuleae species (one representative per genus randomly selected from GenBank), with two *Helianthus* species ( *H. annuus* and *H. devernii*) as outgroups (Table S1). From the plastid genomes of all 23 species, we extracted protein‐coding sequences (CDS) and identified a set of 79 genes consistently present across all 10 *Blumea* species, which were then concatenated into a combined alignment matrix; missing sequences in other taxa were coded as missing data. Alignments were performed using MAFFT (Katoh and Standley 2013), the concatenated dataset was partitioned by gene to accommodate rate heterogeneity across loci. Phylogenetic analyses were conducted using maximum likelihood (ML) and Bayesian inference (BI). ML analysis was performed with IQ‐TREE (v2.0.7) (Minh et al. 2020), using ModelFinder (Kalyaanamoorthy et al. 2017) for best‐fit model selection under BIC and 1000 ultrafast bootstrap replicates for node support. BI was conducted with MrBayes (v3.2.7a) (Ronquist and Huelsenbeck 2003; Ronquist et al. 2012), with models determined by ModelFinder; two MCMC runs were performed for 10 million generations, sampling every 1000 generations with 25% burn‐in, and convergence was confirmed by ASDSF < 0.01. Node support was assessed by posterior probabilities (PP). Resulting trees were visualized with FigTree v1.4.2 (Rambaut 2018).

Based on the results of the phylogenetic tree inferred from the 23 species, the three species most closely related to *Blumea*— *Carpesium abrotanoides* , *Pentanema britannicum*, and *Inula hupehensis*—were selected as closely related outgroups. Of the 79 CDS genes previously identified from the 10 *Blumea* species, 78 were also present in the three outgroups and were retained for downstream analyses. A new ML tree was reconstructed following the same phylogenetic procedures described above to serve as the phylogenetic framework for positive selection analyses. Positive selection signals in the *Blumea* plastomes were detected using the HyPhy v2.5 (Kosakovsky Pond et al. 2005) based on the 78 protein‐coding genes with codon‐based alignments, with the 10 *Blumea* species designated as foreground branches and the three outgroup species as background branches. To comprehensively evaluate positive selection signals, two site‐level methods were employed in parallel: FUBAR (Fast Unconstrained Bayesian AppRoximation) for detecting pervasive positive selection sites (posterior probability ≥ 0.9) (Murrell et al. 2013) and MEME (Mixed Effects Model of Evolution) for detecting episodic positive selection sites (Benjamini‐Hochberg FDR < 0.05) (Murrell et al. 2012).

## 3 Results

### 3.1 Plastid Genome Features

Filtered reads for the six newly assembled *Blumea* plastomes ranged from 24.7 to 37.3 M, with Q20 and Q30 scores of 96.81%–98.77% and 91.83%–96.47%, respectively, and coverage depth ranging from 107.48× to 564.91× (Table 1). The plastomes ranged from 150,783 bp ( *B. sinuata* ) to 151,281 bp ( *B. formosana* ) and all six exhibited the typical quadripartite structure—two inverted repeats (IRs) ranging from 24,924 to 24,957 bp, separated by a large single‐copy region (LSC, 82,524–82,894 bp) and a small single‐copy region (SSC, 18,355–18,509 bp). Overall GC content was nearly constant across species (37.5%–37.6%), with the IRs showing the highest GC content (43%), followed by the LSC (35.6%–35.7%) and SSC (30.8%–31.0%) regions (Table 1; Figure 2). Each plastome contained 131 genes, including 8 ribosomal RNAs, 37 transfer RNAs, and 86 protein‐coding genes, 18 of which were duplicated within the IRs (Table S2). Nine protein‐coding genes and six tRNA genes harbored a single intron, whereas *rps12*, *clpP*, and *ycf3* each contained two introns (Table S2).

**FIGURE 2:**
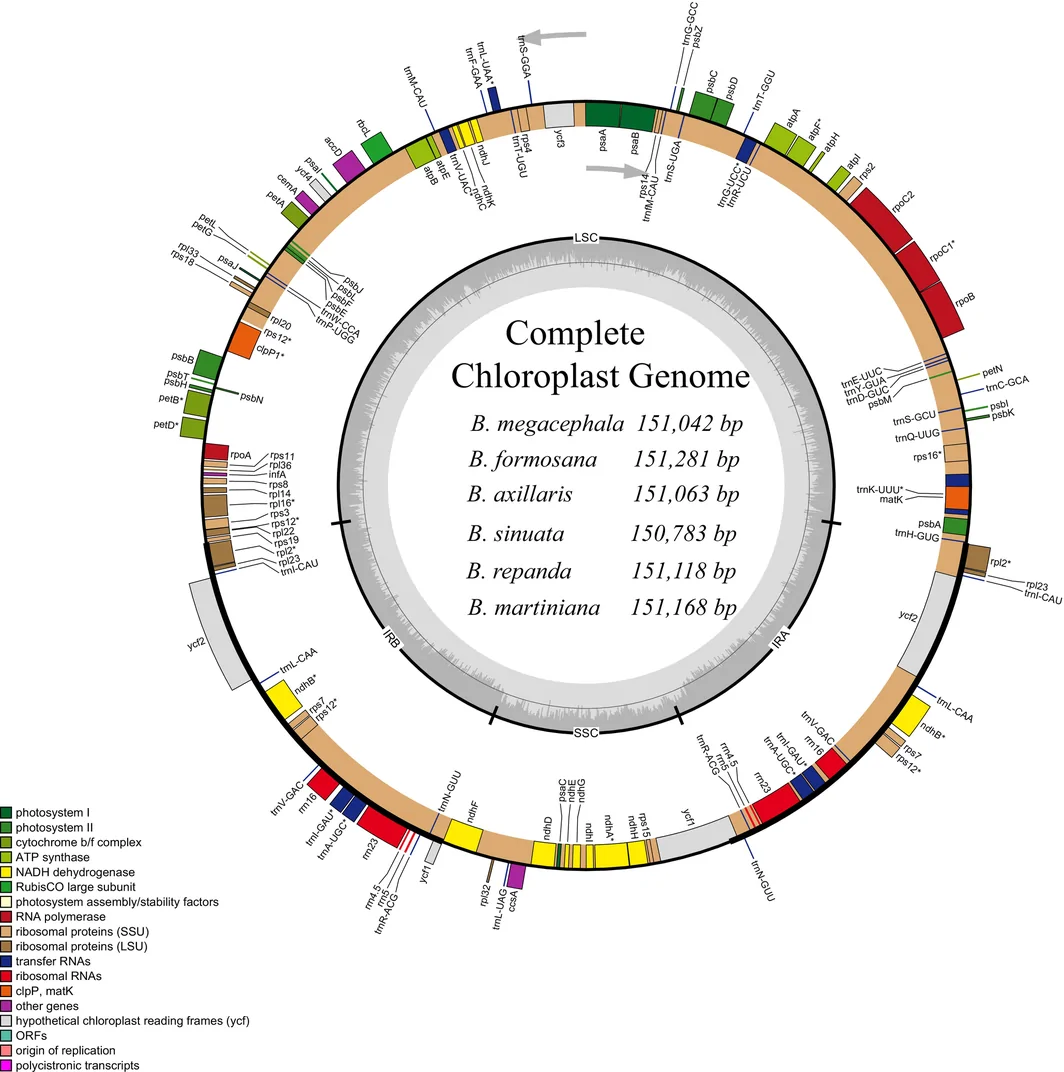
Gene map of six newly sequenced *Blumea* plastomes. Genes on the inside of the large circle are transcribed clockwise and those on the outside are transcribed counterclockwise. The genes are color‐coded based on their functions.

### 3.2 Sequence Divergence Among *Blumea* Species

Gene recombination and arrangement were screened across the 10 *Blumea* plastomes with MAUVE v2.4.0, revealing no detectable rearrangements (Figure S1). Sequence identity relative to *B. martiniana* using mVISTA confirmed IRs to be more conserved than SC regions and coding regions to be more conserved than non‐coding ones (Figure S2); Pi values calculated for all alignments > 300 bp yielded the same pattern. Specifically, in the non‐coding regions, variation spanned 0.19%–8.36% (mean 3.82%), with average values of 4.21% in LSC, 0.87% in IRs, and 6.25% in SSC. Six non‐coding regions showed over 6% variability, including *trnH‐psbA*, *rps16‐trnQ‐UUG*, *atpI‐atpH*, *ycf4‐cemA*, *ndhG‐ndhI*, and *rps15‐ycf1* (Figure 3a). In the coding regions, variation ranged from 0% to 3.70% (mean 1.25%), averaging 1.27% in LSC, 0.37% in IRs, and 1.92% in SSC. Four genes—*ycf1*, *rps8*, *ndhF*, and *matK—*showed the highest variability (Figure 3b). Collectively, non‐coding regions diverged 3.06‐fold more than coding regions, and IR regions were more conserved than SC regions across the 10 complete cp genomes. The results indicated that the evolution of *Blume*a was relatively conservative.

**FIGURE 3:**
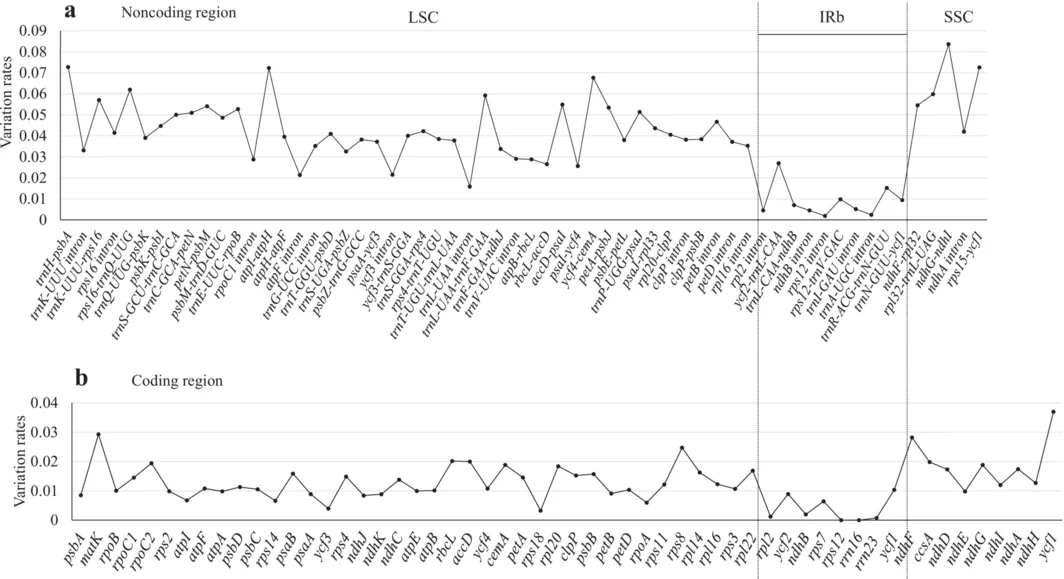
Nucleotide diversity (Pi) across non‐coding (a) and coding (b) regions longer than 300 bp within the 10 *Blumea* plastomes.

### 3.3 Comparisons of the Plastomes in *Blumea*

Comparison of the IR/SC boundary regions across the 10 *Blumea* plastomes revealed generally conserved IR regions, along with limited structural variation at specific junctions (Figure 4). At the LSC/IRb boundary, *rps19* is situated in the LSC region, 10–13 bp from the junction, while *rpl2* resides in IRb, 52–60 bp away. At the IRb/SSC border, a truncated copy of *ycf1* straddles the junction, with 0–21 bp extending into the SSC in nine species; in *B. balsamifera* , this copy is pseudogenized. The *ndhF* gene is located in the SSC, 1–40 bp from the IRb/SSC border, and overlaps with *ycf1* in the SSC of six species (*B. megacephala, B. repanda, B. sinuata, B. axillaris, B. tenella and B. oxyodonta*). The intact *ycf1* copy in the SSC extends 564–581 bp into IRa across the SSC/IRa junction. At the IRa/LSC boundary, *rpl2* and *trnH* are consistently positioned 52–60 bp and 2–4 bp, respectively, from the junction in all 10 plastomes.

**FIGURE 4:**
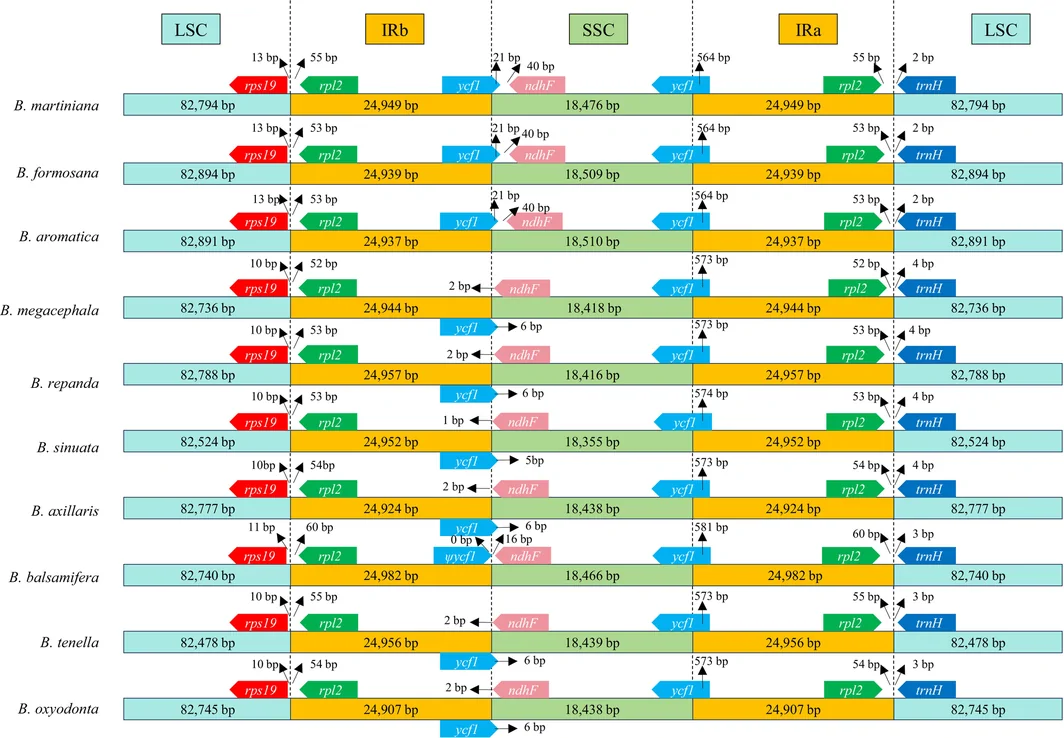
Comparison of the border positions of LSC, SSC, and IR regions in 10 *Blumea* plastomes.

### 3.4 Repeat Sequence Analysis

A total of 395 dispersed repeats were identified across the 10 *Blumea* plastomes, comprising 181 forward, 202 palindromic, 11 reverse, and one complementary repeat (Figure 5a; Table S3). *B. axillaris* exhibited the highest number of repeats (49), while *B. martiniana* had the fewest (34) (Figure 5a). Forward and palindromic repeats were present in all species; reverse repeats were detected in six species, whereas complementary repeats were exclusive to *B. tenella* (Table S3). The majority of dispersed repeats were located in the LSC and IR regions, with only a few in the SSC (Figure S3a; Table S3). Most repeats resided in intergenic spacers (IGS), with minor occurrences in introns or coding sequences (Figure S3b; Table S3). Repeats of 30–39 bp were the most abundant, followed by those of 40–49 bp; repeats exceeding 50 bp were the least frequent (Figure 5b; Table S3). In addition, 79 tandem repeats were identified across the 10 plastomes (Figure S3c; Table S4).

**FIGURE 5:**
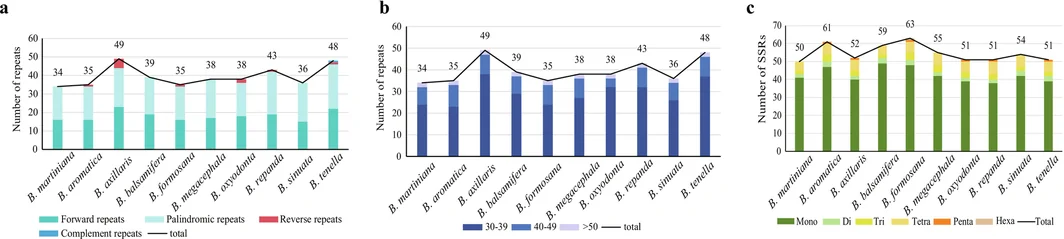
(a) Number of dispersed repeats by repeat types, (b) Number of dispersed repeats by length. (c) Number of simple sequence repeats (SSRs) by repeat types.

A total of 547 simple sequence repeats (SSRs) were identified across the 10 *Blumea* plastomes, with variation in SSR type and abundance among species (Figure 5c; Table S5). Mono‐, di‐, tri‐, and tetranucleotide SSRs were present in all species, whereas pentanucleotide repeats were detected in six species, and hexanucleotide repeats exclusively in *B. formosana* . *B. formosana* exhibited the highest number of SSRs (63), and *B. martiniana* the lowest (50). Mononucleotide repeats were the most abundant (425, 77.70% of all SSRs), followed by tetra‐ (61, 11.15%), tri‐ (29, 5.30%), di‐ (25, 4.57%), penta‐ (6, 1.10%), and hexanucleotide (1, 0.18%) repeats (Figure 5c; Table S5). Among mononucleotide SSRs, A/T motifs overwhelmingly dominated over G/C motifs (417 vs. 8), and AT‐rich composition was also prevalent across other SSR types (Table S5). As expected, the majority of SSRs were located in the LSC region (75.87%), with fewer in the IR (14.26%) and SSC (9.87%) regions (Figure S3d; Table S5). Most SSRs resided in intergenic spacers (65.81%), followed by introns (19.38%) and coding sequences (14.81%) (Figure S3e; Table S5). Within coding regions, SSRs were predominantly distributed in *rpoB*, *rpoA*, *ycf1*, *rpoC1*, *rpoC2*, *psbC*, and *ndhD* (Table S5).

### 3.5 Codon Use Preference Analysis

A total of 52 coding sequences (CDSs) that met the established filtering criteria were selected for codon usage bias analysis. These sequences encoded between 18,281 and 19,764 codons across the 10 *Blumea* species. Among the 52 CDSs, 59 codons exhibited consistent usage preferences across all plastomes. A total of 29 codons showed frequent usage (RSCU > 1), 28 of which ended in A or U; 30 codons were used less frequently (RSCU < 1), 28 of which ended in C or G (Figure 6; Table S6). UUA (Leu) exhibited the highest RSCU value (1.89–1.94), whereas AGC (Ser) showed the lowest (0.32–0.33). Leucine was the most abundant amino acid (2023–2177 codons; 10.97%–11.07%), and cysteine was the least abundant (211–224 codons; 1.12%–1.16%). The most frequently used codon was AUU (Ile, 810–876 copies), and the least frequent was UGC (Cys, 54–65 copies).

**FIGURE 6:**
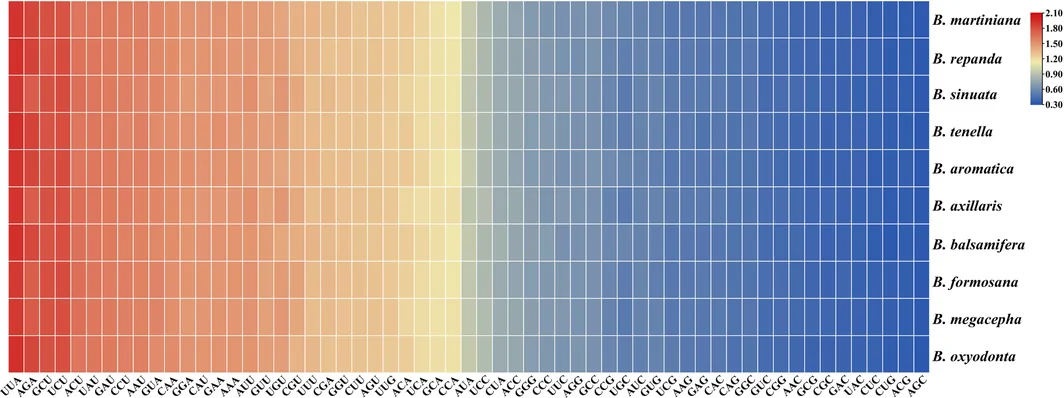
Heat map of RSCU values of protein‐coding genes for 10 *Blumea* plastomes.

### 3.6 Phylogenetic Analysis and Gene Selective Pressure Analysis

Based on 79 shared protein‐coding genes from 23 taxa (including two outgroups), we constructed phylogenetic trees of the Inuleae using maximum likelihood (ML) and Bayesian inference (BI) methods, with two *Helianthus* species as outgroups. In the ML tree (Figure 7), the 21 Inuleae species were divided into four major clades. Clade I comprised only *Tugarinovia mongolica*; Clade II included seven species (*Anaphalis yunnanensis*, *Helichrysum argyrophyllum*, *Gnaphalium polycaulon* , *Filago arvensis* , *Leontopodium sinense*, *Laggera crispata*, and *Pluchea pteropoda*); Clade III comprised three species ( *Carpesium abrotanoides* , *Pentanema britannicum*, and *Inula hupehensis*); and Clade IV was the *Blumea* clade, containing 10 species. All *Blumea* species formed a highly supported monophyletic group, which was internally divided into three subclades. Subclade I comprised six species ( *B. axillaris* , *B. oxyodonta*, *B. megacephala* , *B. repanda* , *B. sinuata* , and *B. tenella* ), within which *B. sinuata* was sister to *B. tenella* , and *B. megacephala* was sister to *B. repanda* ; these four species together formed a clade that was then sister to *B. axillaris* and *B. oxyodonta*. Subclade II consisted of a single species, *B. balsamifera* , which formed a sister lineage to Subclade I. Subclade III comprised three species (*B. aromatica*, *B. formosana* , and *B. martiniana*), with *B. martiniana* as the basal taxon, and *B. aromatica* sister to *B. formosana* . Notably, all three species of Subclade III belong to Section Macrophylla. Additionally, the topology of the 10 *Blumea* species in the BI tree was entirely congruent with that in the ML tree (Figure S4). The remaining taxa showed minor differences, for example, in the BI tree, *Laggera crispata* and *Pluchea pteropoda* clustered with *Carpesium abrotanoides* and its allies, whereas in the ML tree, they clustered with the *Anaphalis yunnanensis* clade.

**FIGURE 7:**
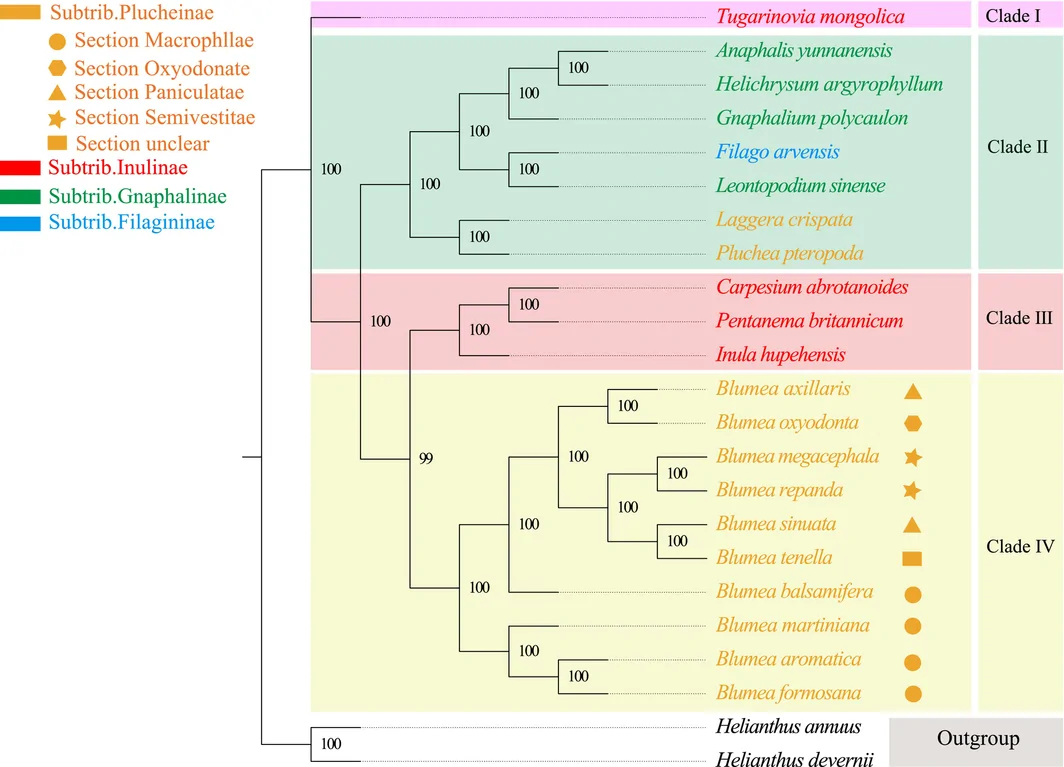
Maximum likelihood (ML) phylogenetic tree of *Blumea* and its allied species based on 79 shared CDS genes. Numbers at nodes represent bootstrap support values (≥ 50%) from 1000 replicates.

To detect positive selection signals in the *Blumea* plastomes, the FUBAR method was first employed to evaluate pervasive positive selection at the site level. However, no significant sites were detected (posterior probability ≥ 0.9) (Table S7), indicating an absence of pervasive positive selection along the *Blumea* lineages. The MEME method was then applied to detect episodic positive selection at the site level, with the 10 *Blumea* species designated as foreground branches. Following FDR correction (FDR < 0.05), a total of 21 genes were identified as containing significant positively selected sites (Table 2), and are considered important candidate genes potentially involved in adaptive evolution of *Blumea*. Among these, *ycf1* (75 sites) contained the highest number of positively selected sites, followed by *ycf2* (17 sites) and *rpoB* (7 sites). These 21 candidate genes are primarily involved in photosynthesis (*psaA*, *psaB*, *psbA*), energy metabolism (*atpA*, *atpF*, *atpI*), gene transcription (*rpoB*, *rpoC1*), and protein synthesis (*rpl20*, *rps12*). These findings suggest that adaptive evolution in *Blumea* may involve coordinated adjustments across multiple functional pathways.

**TABLE 2:** MEME analysis of episodic positive selection in chloroplast genes of *Blumea*.

No.	Gene	Total sites	*Blumea* sites	FDR sites	Positive selection
1	*ycf1*	75	75	75	Yes
2	*ycf2*	17	17	17	Yes
3	*rpoB*	7	7	7	Yes
4	*ndhA*	6	6	6	Yes
5	*matK*	3	3	3	Yes
6	*petA*	3	3	3	Yes
7	*psaB*	2	2	2	Yes
8	*accD*	2	2	2	Yes
9	*ndhF*	2	2	2	Yes
10	*ccsA*	2	2	2	Yes
11	*rpoC1*	2	2	2	Yes
12	*rps12*	2	2	2	Yes
13	*psbA*	1	1	1	Yes
14	*ndhB*	1	1	1	Yes
15	*cemA*	1	1	1	Yes
16	*atpF*	1	1	1	Yes
17	*psaA*	1	1	1	Yes
18	*ndhD*	1	1	1	Yes
19	*ndhG*	1	1	1	Yes
20	*atpA*	1	1	1	Yes
21	*rpl20*	1	1	1	Yes

## 4 Discussion

In this study, we assembled and analyzed six complete chloroplast genomes of *Blumea*, together with four previously published plastomes. All 10 genomes exhibited a highly conserved quadripartite structure—comprising a pair of IRs separating the LSC and SSC regions—consistent with the canonical organization reported in other Asteraceae species (Iram et al. 2019; Jung et al. 2021; Karimov et al. 2025; Kumar et al. 2009; Shen et al. 2017; Sablok et al. 2019; Hatmaker et al. 2020; Kim et al. 2020; Yang et al. 2023; Kim et al. 2024; Duan et al. 2024). The plastid genomes of the six newly sequenced *Blumea* species ranged from 150,783 to 151,281 bp in length, falling within the typical size range observed across most angiosperms (Downie and Palmer 1992; Wicke et al. 2011).

The 10 *Blumea* plastomes (including six newly sequenced and four downloaded from GenBank) each contained 130–131 genes, comprising 8 rRNA genes, 36–37 tRNA genes, and 85–86 protein‐coding genes. Additionally, the gene numbers and orders in the 10 *Blumea* plastomes were highly conserved and no gene rearrangements or inversions were detected. Similar to other plants (Asaf et al. 2017; Liu et al. 2021), GC content was elevated in the IR regions relative to the LSC and SSC regions, which might be related to the existence of rRNA genes known for their high GC content within the IR region. Previous work indicates that the IR region's elevated GC content stems largely from the accumulation of GC‐rich genes, a bias that helps stabilize and conserve the chloroplast genome (Khakhlova and Bock 2006; Wu and Chaw 2015).

IR expansion and contraction are widespread evolutionary events in plant genomes and represent a primary driver of structural variation in chloroplast genomes of certain taxa (Zhu et al. 2016). In this study, the LSC/IRb, SSC/IRa, IRa/LSC borders were consistent across the 10 *Blumea* plastomes. There were slight differences in the IRb/SSC border, which showed an overlap between *ycf1* and *ndhF* in six species but not in the other four species. Studies indicate that genes showing high variability at IR boundaries can be used as evolutionary markers to explore phylogenetic relationships among lineages (Wang et al. 2008). The results of the phylogenetic analysis support the conclusion that the six species with overlapping *ycf1* and *ndhF* genes at the IR/SSC boundary cluster are more closely related. In the remaining four species with non‐overlapping *ycf1* and *ndhF* genes, *B. martiniana*, *B. formosana* , and *B. aromatica* clustered together, while *B. balsamifera* formed an independent clade. In general, the gene sizes and distributions among the 10 *Blumea* species were highly conserved, and the boundary genes (*ycf1* and *ndhF*) can serve as molecular markers in population genetic analysis.

Long repeats are essential for chloroplast‐genome rearrangement and stability (Weng et al. 2014; Cavalier‐smith 2002). We identified 395 dispersed repeats whose number, type, and genome distribution are conserved. Most were 30–39 bp palindromic or forward repeats, a pattern shared with other angiosperms (Karimov et al. 2025; Ren et al. 2018; Ren et al. 2024; Raman and Park 2022). Microsatellites are key for population‐genetic and phylogenetic studies because SSRs ≥ 10 bp readily undergo slipped‐strand mispairing‐the main process generating polymorphisms (Rose and Falush 1998; Raubeson et al. 2007; Huotari and Korpelainen 2012; Zhang, Zhang, et al. 2019; Wang et al. 2020). In this study, 547 microsatellites (50–63) were detected, of which mononucleotide repeats were the most abundant, followed by tetranucleotides, and only one hexanucleotide was present. This number is comparable to that for some Asteraceae species (Iram et al. 2019), but differs from that of other Asteraceae species (Shen et al. 2017; Duan et al. 2024; Curci et al. 2015). Most SSRs were mononucleotide A/T repeats, consistent with earlier reports that plastid SSRs typically comprise short polyA or polyT tracts (Kuang et al. 2011; Martin et al. 2013; Zhou et al. 2018). Similar to other studies (Duan et al. 2024; Eguiluz et al. 2017; Yin et al. 2018), the majority of cpSSRs are detected in non‐coding regions. These cpSSRs are typically short mononucleotide tandem repeats that exhibit intraspecific variations in repeat numbers, which serve as candidate molecular markers for genetic identification.

mVISTA analysis demonstrated substantial sequence conservation among the 10 plastomes. Consistent with expectations, non‐coding regions showed greater variation than coding regions, a pattern corroborated by nucleotide diversity estimates. In addition, high‐resolution markers were also identified based on nucleotide polymorphisms: *trnH‐psbA*, *ycf1*, and *matK* proved to be effective for genus‐level phylogenetic studies (Kumar et al. 2009; Kim et al. 2020; Dong et al. 2015; Dong et al. 2012). In contrast to hypervariable regions, photosynthesis‐ and translation‐related core genes (e.g., *rps12*, *rpl23*) are fully conserved, showing strong functional constraints.

Codons are essential for conveying genetic information as they direct the translation of mRNA into proteins (Qiu et al. 2011). Codon usage bias (CUB), a non‐random choice among synonymous codons, is ubiquitous across organisms and is interpreted as a footprint of evolutionary processes that optimize cellular efficiency and adaptation to environmental conditions (Batzman and Margalit 2011). If third‐position mutations are neutral, synonymous codons should be selected at random, yielding equal GC and AT usage within degenerate codon groups of a gene or genome (Zhang et al. 2007). CUB may be influenced by genetic drift, natural selection, and mutations (Akashi 1997; Chen et al. 2004). In our study, 10 *Blumea* plastomes exhibited identical codon usage patterns, with the majority of preferred codons terminating with A/U, a pattern consistent with numerous angiosperm plastomes (Zhang et al. 2012; Nie et al. 2013; Tyagi et al. 2020). The codons UUA (Leu) and AGC (Ser) exhibited the highest and lowest RSCU values, respectively. Among all amino acids, Leucine showed the highest frequency while Cysteine the lowest—a pattern consistently reported across multiple Asteraceae species (Shen et al. 2017; Nie et al. 2013). In conclusion, comprehensive codon usage analysis provides critical insights into the molecular evolutionary mechanisms underlying *Blumea* plastomes.

As the largest genus in the Inuleae (Anderberg 1991), *Blumea* has been the subject of considerable debate regarding its morphological diversity, phylogenetic position, and character evolution since its initial description. Molecular marker‐based studies (Pornpongrungrueng et al. 2007, 2009) identified two major clades (the *B. densiflora* clade and the *B. lacera* clade) and an independent lineage, *B. balsamifera* ; subsequent studies (Zhang, Yuan, et al. 2019; Chung et al. 2022) have corroborated this framework. Chloroplast genomes are important sources for the development of plant molecular markers (Zhao et al. 2021; Abdullah et al. 2021). In this study, phylogenetic trees reconstructed from 23 taxa of *Blumea* and related species showed that the macro‐topology is highly congruent with previous studies (although the sampling combinations are not entirely identical, no conflicting species placements were observed), and similarly supports the “two clades plus one independent species” framework. Previous studies (Pornpongrungrueng et al. 2007) suggested that the *B. densiflora* clade mainly comprises evergreen woody species, whereas the *B. lacera* clade consists predominantly of annual herbs in open habitats. Although our topology is similar, we did not observe consistent differences in habitat, ecology, or distribution between the two subclades (Flora of China Editorial Committee, 1979). For instance, Subclade I contains both *B. tenella* and *B. axillaris* (annuals) as well as perennials such as *B. megacephala* (Randeria 1960; Flora of China Editorial Committee, 1979); a similar admixture is also observed within Subclade III. This mixing of life forms within subclades suggests that the evolution of life‐history strategies in this genus may be more complex than previously recognized. It is noteworthy that *B. balsamifera* formed an independent lineage in this study, which is consistent with previous molecular marker‐based studies (Pornpongrungrueng et al. 2007). As a widely distributed species with significant medicinal value in this genus, the unique phylogenetic position of *B. balsamifera* may suggest that it has undergone a different evolutionary history from other *Blumea* species, and the possibility that it has retained some ancestral traits cannot be ruled out. Furthermore, the three sampled species of Section Macrophylla (*B. aromatica*, *B. formosana* , and *B. martiniana*) formed a strongly supported monophyletic group in both bootstrap and Bayesian analyses, which is consistent with their traditional placement within this section; the overlap patterns of *ycf1* and *ndhF* at the IR/SSC boundaries were consistent with the inferred topology, suggesting their potential utility as auxiliary structural characters for lineage identification in this genus. It is noteworthy that although ITS has been widely used in this genus, its sequence variation, while informative at the intergeneric level, is insufficient to resolve complex interspecific relationships within the genus (Pornpongrungrueng et al. 2007). Subsequent attempts using the faster‐evolving 5S‐NTS also failed to provide sufficient resolution (Pornpongrungrueng et al. 2009), indicating inherent limitations of such nuclear markers in recent radiations. Moreover, nuclear‐plastid topological conflicts detected at the tribal level (Abdullah et al. 2021; Mandel et al. 2019), resulting from uniparental inheritance and reticulate evolution, further confirm the unreliability of single nuclear genes. Therefore, integration of single‐copy nuclear genes or complete plastome data represents an important approach for obtaining more robust inferences in *Blumea*. In addition, in the ML tree, *Laggera crispata* and *Pluchea pteropoda*, both belonging to the subtribe Plucheinae, did not cluster with *Blumea* (also of Plucheinae), but rather grouped with species of the Gnaphalieae; in the BI tree, they were more closely related to *Carpesium abrotanoides* . This topological instability may be associated with the still unresolved generic delimitation within the Inuleae (Anderberg et al. 2005). Previous studies have suggested that *Laggera* may be polyphyletic, with some species (e.g., *L. decurrens* ) even transferred to the new genus Galgera (Anderberg and Bengtson 2022). Although the taxonomic position of *Laggera* requires further revision, our results generally support a close relationship between *Laggera* and *Pluchea* (Zhou et al. 2022).

In this study, a total of 21 candidate genes were identified as being under positive selection along the *Blumea* lineage, with functions spanning photosynthesis, energy metabolism, gene transcription, and protein synthesis, suggesting that adaptive evolution in *Blumea* may involve coordinated adjustments across multiple functional pathways. Among these candidates, photosynthesis‐related genes (*psaA*, *psaB*, *psbA*, *petA*, *ccsA*, *ndhA*, *ndhB*, *ndhD*, *ndhF*, *ndhG*) and energy metabolism‐related genes (*atpA*, *atpF, accD*) accounted for the largest proportion. Photosystem I (*psaA*, *psaB*) and photosystem II (*psbA*) are core components of the photosynthetic electron transport chain, and the coordinated positive selection of ATP synthase (*atpA*, *atpF*) and the cytochrome b6f complex (*petA*) supports the overall optimization of photosynthetic electron transport and energy metabolism. Multiple NADH dehydrogenase complex genes (*ndhA*, *ndhB*, *ndhD*, *ndhF*, *ndhG*) and the acetyl‐CoA carboxylase gene (*accD*) are involved in cyclic electron transport and fatty acid synthesis, respectively, and their functions are closely related to photosynthetic adaptability in *Blumea*. *Blumea* species are predominantly distributed in tropical and subtropical open habitats, exhibiting strong adaptability to varying light environments, and their relatively high photosynthetic efficiency and broad light adaptation range are consistent with the positive selection observed in these photosynthesis‐related genes, implying that *Blumea* may have undergone multi‐level adaptive adjustments in photosynthetic metabolic pathways. This result is consistent with previous observations of positive selection acting on photosynthesis‐related genes (Yang et al. 2020; Fang et al. 2024; Cauz‐Santos et al. 2025; Zheng et al. 2025), indicating that optimization of photosynthetic pathways represents an important direction of plant adaptive evolution. Positive selection signals were also detected on the *Blumea* lineage for *rpoB* and *rpoC1* (transcription), *matK* (RNA processing), as well as *rpl20* and *rps12* (translation), suggesting that the plastid gene expression system—from transcription and RNA processing to translation—may have undergone coordinated adjustments. These genes have also been identified in other angiosperm lineages (Hao et al. 2010; Xiong et al. 2023; Li et al. 2021), indicating that although these genes encode functionally relatively conserved proteins, they continue to experience positive selection across different taxa. Additionally, *cemA* may promote inorganic carbon uptake by regulating proton efflux, and its positive selection may be associated with photosynthetic efficiency or light adaptation, with similar reports in other lineages (Li et al. 2025; Zhao et al. 2026). *ycf1* and *ycf2* encode key components of the plastid inner envelope protein transport complex, with Ycf1 (TIC214) potentially serving as the protein translocation channel and Ycf2 providing ATPase activity to energize the transport process, and these two genes are thought to cooperatively drive the transmembrane translocation of precursor proteins. Positive selection in these two genes has been reported in multiple Asteraceae lineages (Abdullah et al. 2025; Yan et al. 2026; Cho et al. 2024), and they harbored the highest number of positively selected sites in *Blumea* (75 and 17 sites, respectively), suggesting that they may play important roles in the adaptive evolution of *Blumea* and even the Asteraceae, warranting further investigation. The FUBAR analysis detected no pervasive positive selection sites, indicating that the signals described above represent lineage‐specific events along the *Blumea* branches, consistent with the evolutionary model in which plastid genomes are predominantly shaped by purifying selection, with adaptive signals occurring episodically. Overall, adaptive evolution in *Blumea* plastomes is primarily characterized by lineage‐specific episodic positive selection across multiple functional pathways, which may have jointly promoted adaptive differentiation in diverse habitats.

## 5 Conclusion

In this study, we assembled the complete chloroplast genomes of six *Blumea* species and, together with four published genomes, performed comparative genomic and phylogenetic analyses. All 10 plastomes were highly conserved in gene order, size, structure, and GC content, with no rearrangements detected. A total of 395 dispersed repeats, 79 tandem repeats, and 547 SSRs were identified. Codon usage revealed a strong preference for A/U‐ending codons. Six non‐coding and four coding regions were identified as candidate DNA barcodes. Episodic positive selection was detected in 21 genes. Phylogenetic analyses recovered *Blumea* as a highly supported monophyletic group with three subclades: subclade III comprising *B. aromatica*, *B. formosana* , and *B. martiniana* (all belonging to Section Macrophylla), subclade II ( *B. balsamifera* ), and subclade I (the remaining six species). This topology supports the previously recognized “two subclades plus one independent lineage” framework; however, the mixed life forms within subclades suggest more complex ecological adaptive evolution than previously thought. These results provide a genomic foundation for studies on the phylogeny and adaptive evolution of *Blumea*, and the identified SSRs and candidate barcodes may facilitate molecular marker development and population genetics. Nevertheless, our sampling is limited; given the limited resolution of nuclear markers (ITS, 5S‐NTS) in resolving recent radiations, future incorporation of more representative species and nuclear genomic or transcriptomic data will enable a more comprehensive understanding of the phylogenetic framework and adaptive evolution of this genus.

## Author Contributions

**Yuemei Zhao:** formal analysis (lead), methodology (lead), project administration (equal), writing – original draft (lead). **Weixiang Yang:** software (lead). **Changjiang Qian:** resources (lead). **Tao Zhou:** resources (equal). **Xiao Zhang:** funding acquisition (equal). **Xiaodan Chen:** funding acquisition (equal). **Rongxiang Zhang:** formal analysis (equal). **Xun Liu:** supervision (lead), writing – review and editing (lead).

## Funding

This research was funded by the Innovation Capability Support Program of Shaanxi (no. 2025ZC‐KJXX‐119); Natural Science Basic Research Program of Shanxi (no. 202203021212400); Natural Science Basic Research Program of Shanxi Normal University (no. JCYJ2023001); Natural Science Foundation of Guizhou Education University, China (no. 2026GCC010).

## Conflicts of Interest

The authors declare no conflicts of interest.

## Supporting information


**Figure S1:** Mauve alignment of 10 *Blumea* plastomes. Within each of the alignment, local collinear blocks are represented by blocks of the same color connected by line.

**Figure S2:** Sequence identity plot of the 10 *Blumea* plastomes using *B. martinian* as a reference.

**Figure S3:** (a) Number of dispersed repeats in LSC, SSC, and IR regions. (b) Number of dispersed repeats in IGS, CDS, and intron regions. (c) Number of tandem repeats in LSC, SSC and IR regions. (d) Number of SSRs in LSC, SSC, and IR regions. (e) Number of SSRs in IGS, CDS, and intron regions.

**Figure S4:** Bayesian inference (BI) phylogenetic tree of *Blumea* and its allied species based on 79 shared CDS genes. Numbers at nodes represent posterior probabilities (≥ 0.50).

**Table S1:** List of species and their accession numbers in GenBank included in the phylogenetic analyses of complete chloroplast genomes.
**Table S2:** List of genes in the plastome of 10 *Blumea* species.
**Table S3:** The Dispersed repeat sequences distribution in the 10 *Blumea* plastid genomes.
**Table S4:** List of tandem repeats distribution in the 10 *Blumea* plastid genomes.
**Table S5:** Simple sequence repeats (SSRs) in the 10 *Blumea* plastid genomes.
**Table S6:** Codon usage and relative synonymous codon usage (RSCU) values of protein‐coding genes of 10 *Blumea* species.
**Table S7:** FUBAR analysis of positive selection in 78 chloroplast genes of 10 *Blumea* species.

## Data Availability

The complete chloroplast genome sequences of six *Blumea* species have been submitted into NCBI with accession numbers PX404818–PX404823; the associated BioProject number is PRJNA1334118.
