# Supplementary material for: Plastomes of Ten Blumea Species (Asteraceae): Comparative Analysis and Phylogenetic Relationships
Source: Ecol Evol. 2026 Oct 12;16(10):e74361. doi: 10.1002/ece3.74361 (PMC13658934; doi:10.1002/ece3.74361)
Supplement: Supplementary file 1 [file ECE3-16-e74361-s001.pdf]

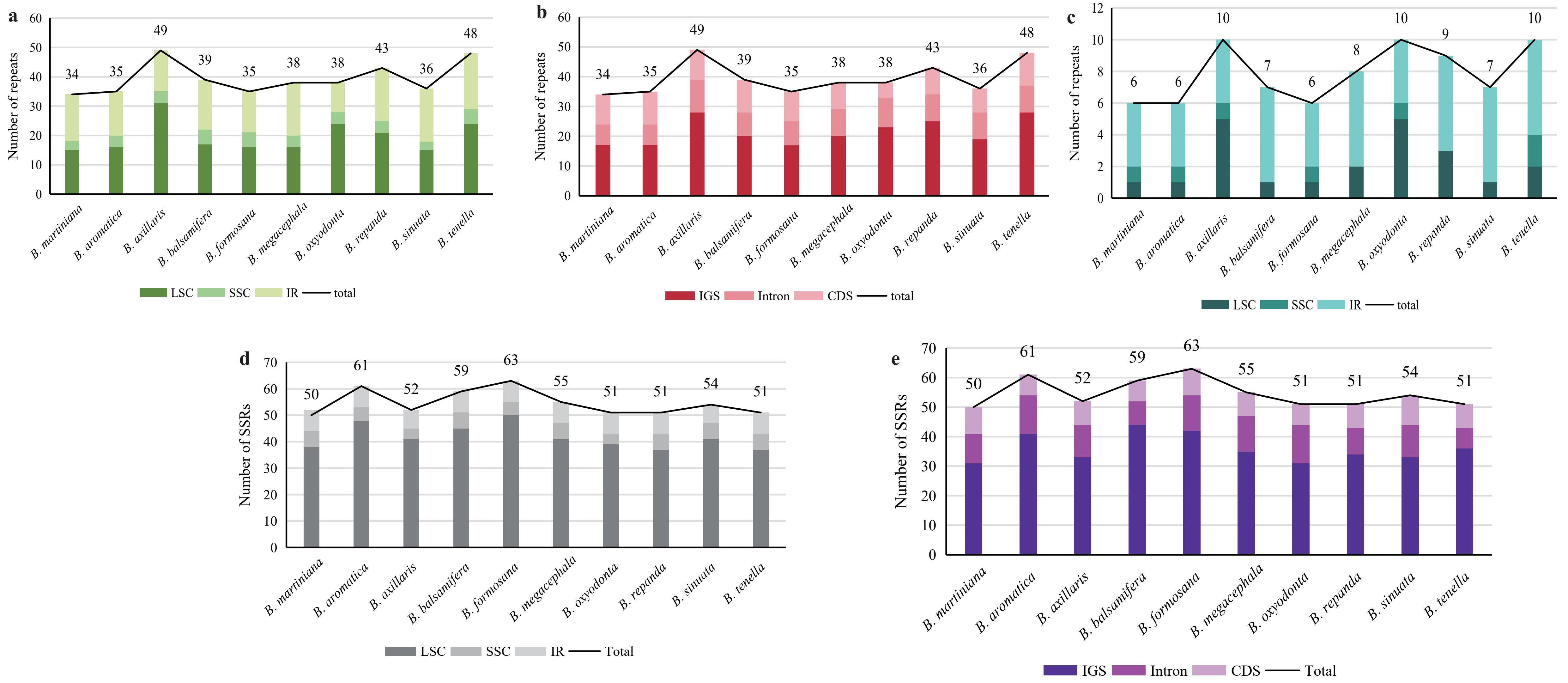
a
Number of repeats
34
35
49
39
35
38
38
43
36
48
B. martiniana
B. aromatica
B. axillaris
B. balsamifera
B. formosana
B. megacephala
B. oxyodonta
B. repanda
B. sinuata
B. tenella
LSC
SSC
IR
total
b
Number of repeats
IGS
Intron
CDS
total
c
Number of repeats
6
6
10
7
6
8
10
9
7
10
LSC
SSC
IR
total
d
Number of SSRs
50
61
52
59
63
55
51
51
54
51
LSC
SSC
IR
Total
e
Number of SSRs
IGS
Intron
CDS
Total
