# Supplementary material for: Plastomes of Ten Blumea Species (Asteraceae): Comparative Analysis and Phylogenetic Relationships
Source: Ecol Evol. 2026 Oct 12;16(10):e74361. doi: 10.1002/ece3.74361 (PMC13658934; doi:10.1002/ece3.74361)
Supplement: Supplementary file 2 [file ECE3-16-e74361-s003.pdf]

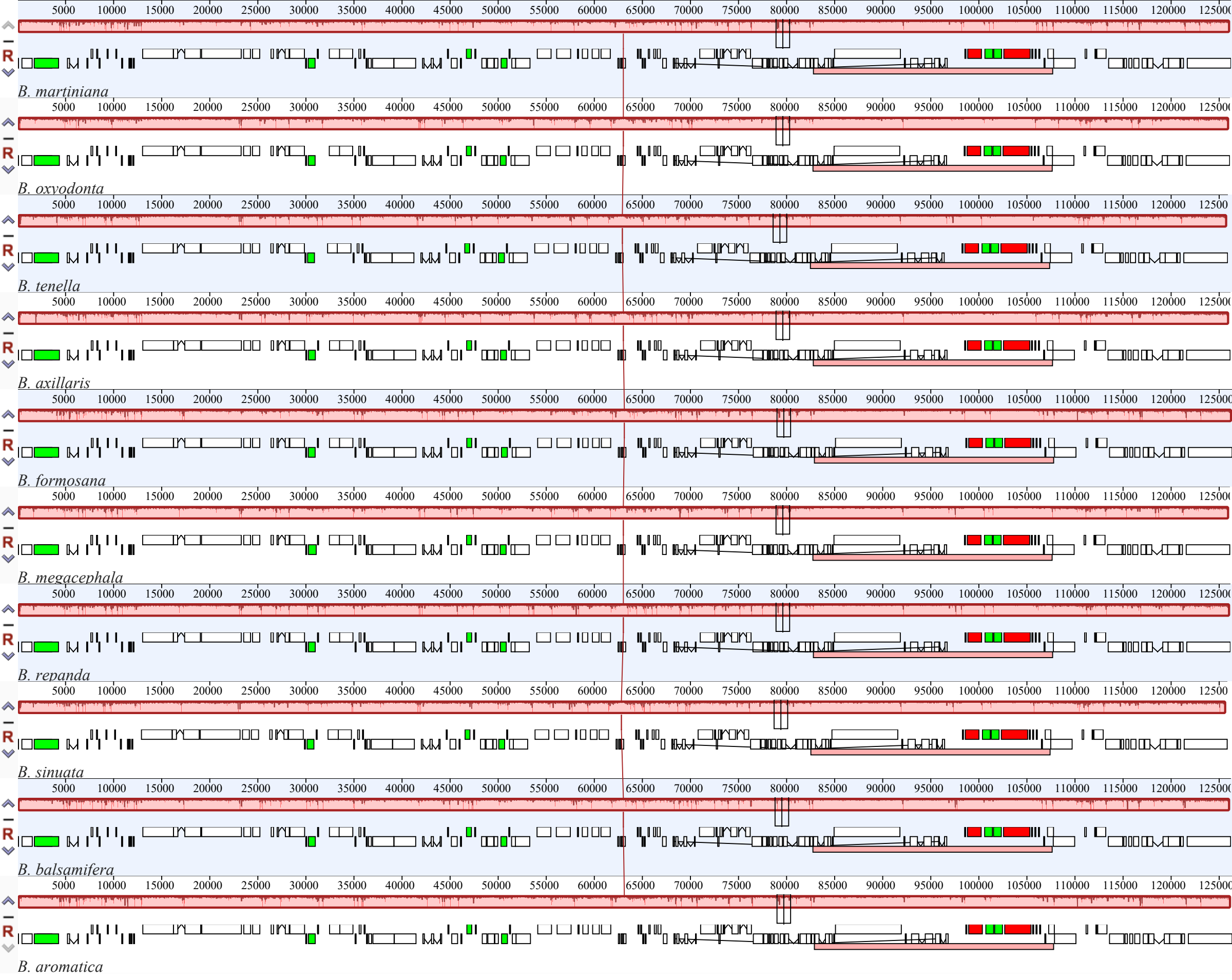
5000 10000 15000 20000 25000 30000 35000 40000 45000 50000 55000 60000 65000 70000 75000 80000 85000 90000 95000 100000 105000 110000 115000 120000 125000
B. martiniana
B. oxyodonta
B. tenella
B. axillaris
B. formosana
B. megacephala
B. repanda
B. sinuata
B. balsamifera
B. aromatica
