# Supplementary material for: Plastomes of Ten Blumea Species (Asteraceae): Comparative Analysis and Phylogenetic Relationships
Source: Ecol Evol. 2026 Oct 12;16(10):e74361. doi: 10.1002/ece3.74361 (PMC13658934; doi:10.1002/ece3.74361)
Supplement: Supplementary file 3 [file ECE3-16-e74361-s004.pdf]

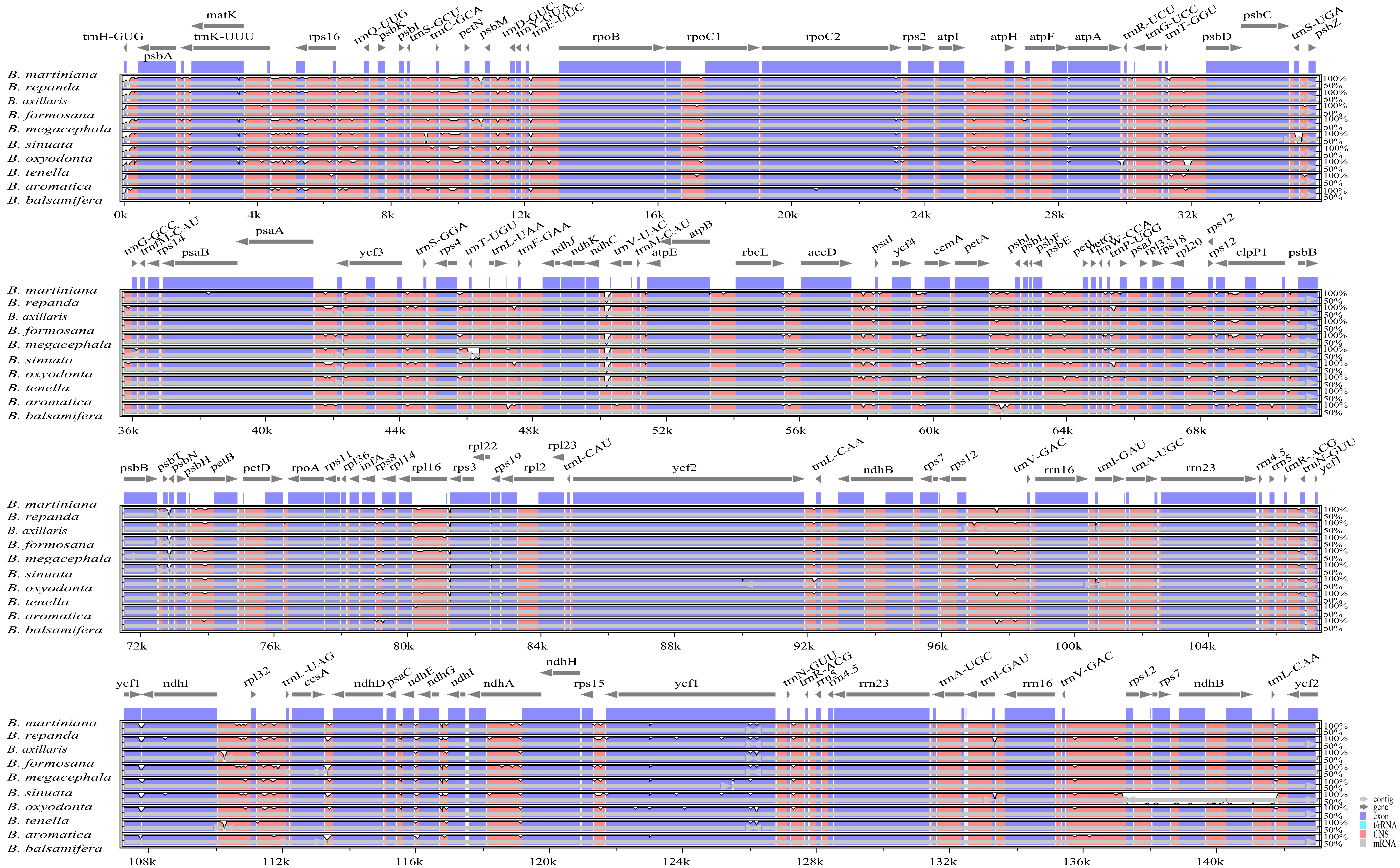

B. martiniana
B. repanda
B. axillaris
B. formosana
B. megacephala
B. sinuata
B. oxyodonta
B. tenella
B. aromatica
B. balsamifera
100%
50%
0k
4k
8k
12k
16k
20k
24k
28k
32k
36k
40k
44k
48k
52k
56k
60k
64k
68k
72k
76k
80k
84k
88k
92k
96k
100k
104k
108k
112k
116k
120k
124k
128k
132k
136k
140k
trnH-GUG
psbA
matK
trnK-UUU
rps16
trnQ-UUG
psbK
psbI
trnS-GCU
trnC-GCA
petN
psbM
trnD-GUC
trnY-GUA
trnE-UUC
rpoB
rpoC1
rpoC2
rps2
atpI
atpH
atpF
atpA
trnR-UCU
trnG-UCC
trnT-GGU
psbD
psbC
trnS-UGA
psbZ
trnG-GCC
trnfM-CAU
rps14
psaB
psaA
ycf3
trnS-GGA
rps4
trnT-UGU
trnL-UAA
trnF-GAA
ndhJ
ndhK
ndhC
trnV-UAC
trnM-CAU
atpB
atpE
rbcL
accD
psaI
ycf4
cemA
petA
psbJ
psbL
psbF
psbE
petL
petG
trnW-CCA
trnP-UGG
psaJ
rpl33
rps18
rpl20
rps12
clpP1
psbB
psbT
psbN
psbH
petB
petD
rpoA
rps11
rpl36
infA
rps8
rpl14
rpl16
rps3
rpl22
rps19
rpl2
rpl23
trnI-CAU
ycf2
trnL-CAA
ndhB
rps7
trnV-GAC
rrn16
trnI-GAU
trnA-UGC
rrn23
rrn4.5
rrn5
trnR-ACG
trnN-GUU
ycf1
ndhF
rpl32
trnL-UAG
ccsA
ndhD
psaC
ndhE
ndhG
ndhI
ndhA
ndhH
rps15
contig
gene
exon
t/rRNA
CNS
mRNA
