# Supplementary material for: Plastomes of Ten Blumea Species (Asteraceae): Comparative Analysis and Phylogenetic Relationships
Source: Ecol Evol. 2026 Oct 12;16(10):e74361. doi: 10.1002/ece3.74361 (PMC13658934; doi:10.1002/ece3.74361)
Supplement: Supplementary file 4 [file ECE3-16-e74361-s005.pdf]

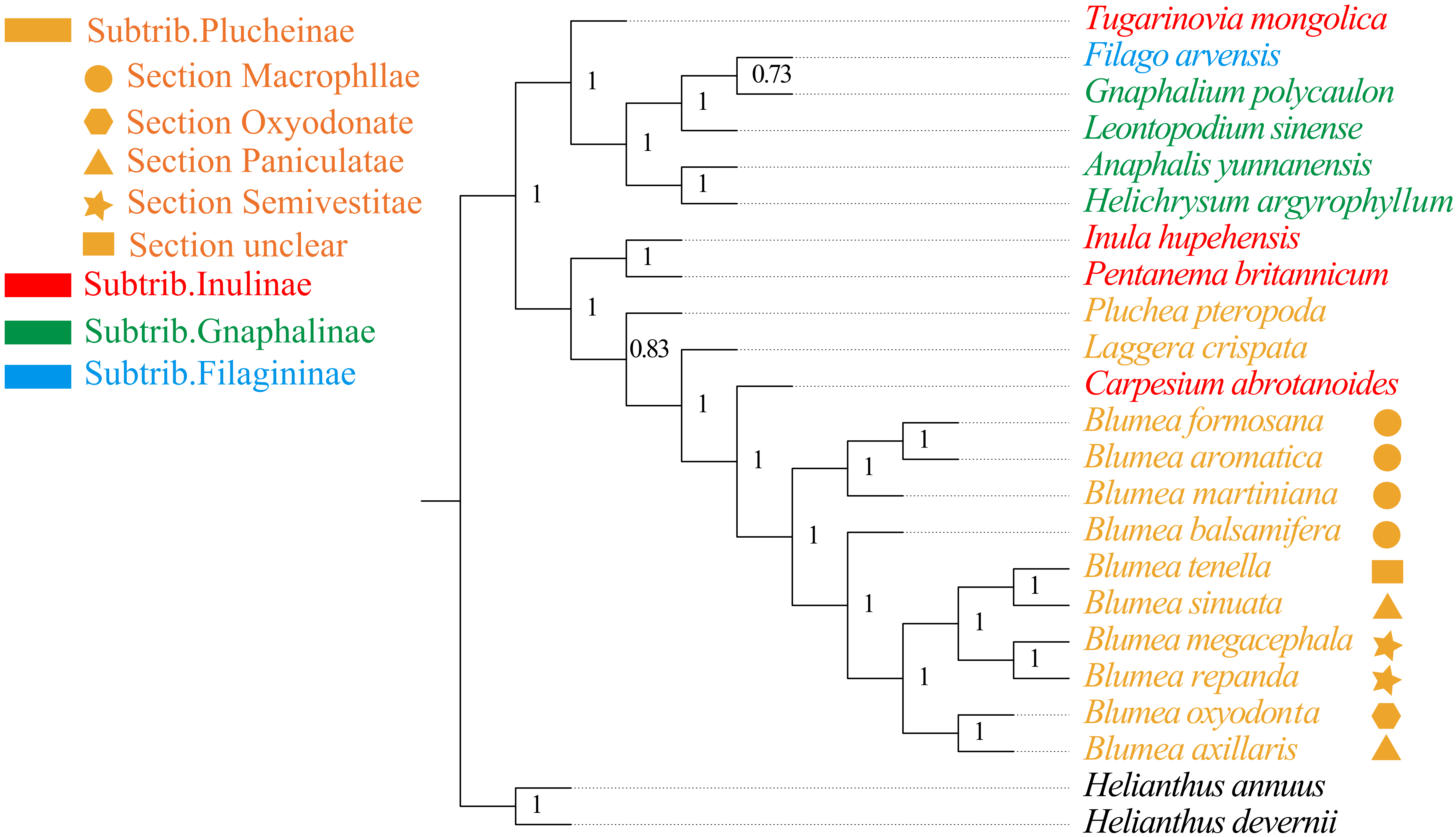
Subtrib.Plucheinae
Section Macrophllae
Section Oxyodonate
Section Paniculatae
Section Semivestitae
Section unclear
Subtrib.Inulinae
Subtrib.Gnaphalinae
Subtrib.Filagininae
Tugarinovia mongolica
Filago arvensis
Gnaphalium polycaulon
Leontopodium sinense
Anaphalis yunnanensis
Helichrysum argyrophyllum
Inula hupehensis
Pentanema britannicum
Pluchea pteropoda
Laggera crispata
Carpesium abrotanoides
Blumea formosana
Blumea aromatica
Blumea martiniana
Blumea balsamifera
Blumea tenella
Blumea sinuata
Blumea megacephala
Blumea repanda
Blumea oxyodonta
Blumea axillaris
Helianthus annuus
Helianthus devernii
0.73
0.83
